# The Letters of a Layman

**Published:** 1921-07-16

**Authors:** 


### THE EDITOR'S LETTER-BOX.

**Correspondence on all subjects is invited, but we cannot in any way be responsible for the opinions expressed by our correspondents, who must give their name and address as a guarantee of good faith, but not necessarily for publication. Correspondents are reminded that brevity of style and conciseness of statement greatly facilitate early insertion.**

### Doctor and Patient.

### "The Letters of a Layman."

*To the Editor of* The Hospital.

Sir,—Your incisive and always stimulating correspondent "Ierne" is advocating the monthly or quarterly resort of every individual to his family doctor in order that the earliest beginnings of ill-health may be detected, and cure or prevention undertaken when there is the highest likelihood of success. It is possible that in a few generations from now such a course will be common—nay, even compulsory. But "Ierne" is ahead of the age —the medical age, I mean—that we live in. Not even the wisest in the medical profession—and most certainly not the rank and file—are yet sufficiently instructed to make this suggested course useful. The beginnings of disease, the prevention of disease, *in the individual* are fruitful subjects for research—nay, are already engaging some of the acutest brains in the profession. But such research is as much in its infancy as the science of public health, the prevention of disease *in the multitude or community,* was a century ago. "Ierne" sees a vision, an ideal, that will be practicable in time—not in my time, possibly not in "Ierne's," but perhaps in that of our grandchildren. Let us hasten it, if we can, let us welcome it when it comes; but do not let us deceive ourselves that it has come yet.—Yours faithfully, Grey Hairs.

